# Auditory and Somatosensory P3 Are Complementary for the Assessment of Patients with Disorders of Consciousness

**DOI:** 10.3390/brainsci10100748

**Published:** 2020-10-17

**Authors:** Jitka Annen, Isabella Mertel, Ren Xu, Camille Chatelle, Damien Lesenfants, Rupert Ortner, Estelle A.C. Bonin, Christoph Guger, Steven Laureys, Friedemann Müller

**Affiliations:** 1 GIGA Consciousness, Coma Science Group, University of Liege, 4000 Liege, Belgium; camillechatelle@gmail.com (C.C.); estelle.bonin@uliege.be (E.A.C.B.); Steven.Laureys@uliege.be (S.L.); 2 Centre du Cerveau (C2), University Hospital Liege, 4000 Liege, Belgium; 3 Schoen Klinik Bad Aibling, 83043 Bad Aibling, Germany; isabella.mertel@gmail.com (I.M.); Fmueller@Schoen-Kliniken.de (F.M.); 4 Department of Clinical Psychology, University of Tuebingen-, 72074 Tuebingen, Germany; 5 Guger Technologies OG, 8020 Graz, Austria; xu@gtec.at (R.X.); guger@gtec.at (C.G.); 6 Laboratory for NeuroImaging of Coma and Consciousness—Department of Neurology, Massachusetts General Hospital, Harvard Medical School, Boston, 02114 MA, USA; 7 Experimental Oto-rino-laryngology, Department of Neuroscience, Katholieke Universiteit Leuven, 3000 Leuven, Belgium; Damien.Lesenfants@uliege.be; 8 g.tec Medical Engineering, 08038 Barcelona, Spain; ortner@gtec.at; 9 g.tec Medical Engineering GmbH, 4521 Schiedlberg, Austria

**Keywords:** multisensory stimulation, P3, vibrotactile, auditory evoked potentials, disorders of consciousness

## Abstract

The evaluation of the level of consciousness in patients with disorders of consciousness (DOC) is primarily based on behavioural assessments. Patients with unresponsive wakefulness syndrome (UWS) do not show any sign of awareness of their environment, while minimally conscious state (MCS) patients show reproducible but fluctuating signs of awareness. Some patients, although with remaining cognitive abilities, are not able to exhibit overt voluntary responses at the bedside and may be misdiagnosed as UWS. Several studies investigated functional neuroimaging and neurophysiology as an additional tool to evaluate the level of consciousness and to detect covert command following in DOC. Most of these studies are based on auditory stimulation, neglecting patients suffering from decreased or absent hearing abilities. In the present study, we aim to assess the response to a P3-based paradigm in 40 patients with DOC and 12 healthy participants using auditory (AEP) and vibrotactile (VTP) stimulation. To this end, an EEG-based brain-computer interface was used at DOC patient’s bedside. We compared the significance of the P3 performance (i.e., the interpretation of significance of the evoked P3 response) as obtained by ‘direct processing’ (i.e., theoretical-based significance threshold) and ‘offline processing’ (i.e., permutation-based single subject level threshold). We evaluated whether the P3 performances were dependent on clinical variables such as diagnosis (UWS and MCS), aetiology and time since injury. Last we tested the dependency of AEP and VTP performances at the single subject level. Direct processing tends to overestimate P3 performance. We did not find any difference in the presence of a P3 performance according to the level of consciousness (UWS vs. MCS) or the aetiology (traumatic vs. non-traumatic brain injury). The performance achieved at the AEP paradigm was independent from what was achieved at the VTP paradigm, indicating that some patients performed better on the AEP task while others performed better on the VTP task. Our results support the importance of using multimodal approaches in the assessment of DOC patients in order to optimise the evaluation of patient’s abilities.

## 1. Introduction

Following a severe brain injury, patients may fall into a coma. Some of these patients evolve into a state with decreased awareness in the presence of eye opening, referred to as disorders of consciousness (DOC). In this state, patients can either only present reflexive behaviour without signs of awareness (unresponsive wakefulness syndrome, UWS [1], also referred to as vegetative state [2]), or present minimal signs of awareness (minimally conscious state, MCS [3]). Patients who recover functional object use or communication are considered as emerged from the MCS (EMCS), even though cognitive deficits are still common [3]. The differential diagnosis of patients with DOC in clinical practice is primarily based on structured behavioural assessments such as the Coma Recovery Scale-Revised (CRS-R) [4]. The risk of misdiagnosis due to arousal fluctuation can be reduced to some extent by repeating the measurements over time [5]. However, a limitation of the behavioural assessment is that it is highly dependent on motor and language abilities. The vast majority of DOC patients suffers from visual, auditory or motor limitations (e.g., spasticity in 88–96% of DOC patients [6,7]) that can impede their behavioural evaluation [8].

Bedside evaluation of behavioural responsiveness and laboratory/para-clinical investigation via neuroimaging or neurophysiological assessment lead to contradictory results in a considerable amount of patients [9]. Indeed, some patients may show preserved residual brain function as measured with neuroimaging, suggesting a level of cognitive function and consciousness more preserved than what is observable at bedside (e.g., referred to as MCS*) [10,11,12]. Some patients show a dissociation between the absence of command following at bedside and the presence of covert command following (i.e., modulation of brain activity in response to a command; referred to as or cognitive motor dissociation) [13]. A minority of patients who can produce a goal-oriented response to simple instructions, could use these voluntary alterations of brain function for communication purposes with a brain computer interface (BCI) (e.g., referred to as functional locked-in [14]) [15,16].

The limitations of clinical assessment of DOC patients highlight the need for additional diagnostic measurements in clinical routine. These assessments should work independently from behavioural responses, directly probing cerebral resting state activity or task-evoked cerebral responses. Electroencephalography (EEG) is cost-effective, requires limited preparation time, is mobile and easily applicable at the patient’s bedside. Another advantage is its high temporal resolution, making it especially suitable for brain-computer interface (BCI) applications. Yet, it is still difficult to transpose the use of EEG-BCI from healthy subjects to patients with DOC due to high false-negative and false-positive rates [17,18,19].

Event related potentials (ERPs) reflect the (averaged) electrophysiological response to certain sensory, cognitive, or motor events. Simple cortical responses (e.g., P1, N1, P2, P3a) could occur regardless of one’s level of consciousness, and are more likely when the baseline EEG shows activity above 4 Hz [20]. One of the most commonly used ERPs is the P3. It is defined as a positive deflection in the ongoing local field potential about 300 ms after a deviant stimulation in a train of standard stimuli. The P3 can be divided into an early P3a response with a frontal topography reflecting novelty and is unrelated to consciousness and can be present during sleep, coma and UWS ([21,22] for review). The later P3b response over the parietal cortex and is related to cognitive processing [23], is modulated by conscious attention [24,25], and therefore has been proposed as an objective marker of conscious awareness. At the group level, a significant P3 (i.e. not differentiated between the P3a and P3b) response is less commonly observed in UWS than in MCS patients [20]. MCS patients show a higher P3 amplitude in response to their own name compared to unfamiliar names, and following the instruction to count the deviant (active paradigm) as compared to measurements without instruction (passive paradigm) [26].

The P3 can be elicited by, amongst others, auditory [25] and somatosensory [27] stimuli. Given that DOC patients suffered from severe brain injury, it is possible that either of these stimuli cannot be processed. This would impede to evaluate the P3b responses to that modality, even if the patient might be conscious, and can therefore not be used reliably to probe signs of conscious attention. Previous P3 (or even hybrid BCI [28]) studies have however focused only on the brain response to a single sensory stimulus, impeding assessment of patients with limitations in the processing of certain modalities.

This multicentre study includes DOC patients and healthy participants from Liege (LI, Belgium) and Bad Aibling (BA, Germany). We tested if the interpretation of P3 significance (i.e., P3 performance) were equally reliable during direct (i.e., using a theoretical significance threshold) and offline (i.e., using a single subject permutation based significance threshold) processing, and dependent on diagnosis, aetiology and time since injury. Second, we tested whether the P3 performance to the auditory and tactile stimulation differed within participants to evaluate if multimodal assessment provides additional information over unimodal assessment.

## 2. Materials and Methods

### 2.1. Participants

Participants in this multicentre study were recruited in the University Hospital of Liège, Belgium (LI) and in the Schoen Klinik, Bad Aibling, Germany (BA). The study included a convenience sample of 58 DOC patients (31 from LI, 27 from BA) and 15 healthy participants (7 from LI and 8 from BA). Patients suffered either from a traumatic or non-traumatic (e.g., haemorrhage, stroke, anoxia) brain injury. Time elapsed since injury was at least one month before study enrolment. All patients were spontaneously breathing and had no history of neurological or mental disease prior the event of brain injury. Patients with known auditory deficits or open wounds following craniotomy were excluded from the study.

Patients were evaluated several times with the CRS-R [29]. Precisely, the patients in LI were assessed five times within one week of the BCI assessment including once on the day of the BCI assessment; the patients in BA were repeatedly tested every two weeks before study enrolment and retested on the day of BCI assessment. CRS-R examinations were performed by experienced and trained assessors. The diagnosis for each patient was given as the best diagnosis out of the series of clinical tests, in order to account for vigilance fluctuations [5]. Clinical, demographic and P3 data are summarised in Table 1. The study was approved by the ethical committees of the University of Tuebingen and the University hospital of Liège. Written informed consent was obtained from the patients’ legal representatives and from the healthy participants.

### 2.2. P3 Assessment and Data Processing

In the present study, we used an auditory and a vibrotactile P3 paradigm from a commercially available EEG-based BCI system (mindBEAGLE, g.tec medical engineering, Austria). All settings of mindBEAGLE, as described in this section, are preconfigured and cannot be tuned by users. P3 assessments were performed at the patient’s bedside. If the patient showed prolonged eye closure before or during the assessment, the paradigm was paused and the patient was aroused (using the CRS-R arousal facilitation protocol) before the start or continuation of the assessment. The assessment consisted of two oddball paradigms: auditory-evoked potentials (AEP) and vibrotactile-evoked somatosensory potentials (VTP). For both active paradigms, the patient was instructed to mentally count the number of deviant stimuli (i.e., the high pitch tones or attended hand receiving vibrations less frequently). In both paradigms, a total of 480 stimuli (of which 60 deviant stimuli) were delivered.

For the AEP paradigm, insert earphones were used to deliver the deviant and the standard stimulus binaurally. Both stimuli had a duration of 100 ms, and the onset-to-onset inter-stimulus interval was 900 ms. The rare (probability of 0.125) deviant stimulus was a pure tone burst with a base frequency of 1000 Hz. The frequent (probability of 0.875) standard stimulus was a pure tone burst with a base frequency of 500 Hz. The AEP paradigm had a total duration of 8 min.

For the VTP paradigm, mechanical vibrating tactors were fixed to the patient’s left and right wrists. The vibrotactile stimuli were delivered by the g.STIMbox (g.tec, Austria) and had a duration of 30 ms and a frequency of 225 Hz with an onset-to-onset inter-stimulus interval of 270 ms. The tactors on one wrist delivered the rare (probability of 0.125) deviant stimulus and the tactor on the other wrist delivered the frequent (probability of 0.875) standard stimulus. In patients from BA, the tactor that delivered the deviant stimulus was applied on the left wrist and the tactor that delivered the standard stimulus on the right wrist, which was reversed in patients from LI. The VTP paradigm had a total duration of 2.4 min.

The EEG was recorded by eight active gel electrodes (g.SCARABEO Ag/AgCl electrodes, g.tec, Austria) placed according to the international 10–20 electrode system at FCz, C3, Cz, C4, CP1, CPz, CP2 and Pz. The ground electrode was on the forehead (AFz) and to the reference was on the right earlobe (BA) or mastoid (LI). Data were recorded at 256 Hz, and band-pass filtered between 0.01 Hz and 30 Hz with a 4th order Butterworth filter.

**Figure 1:**
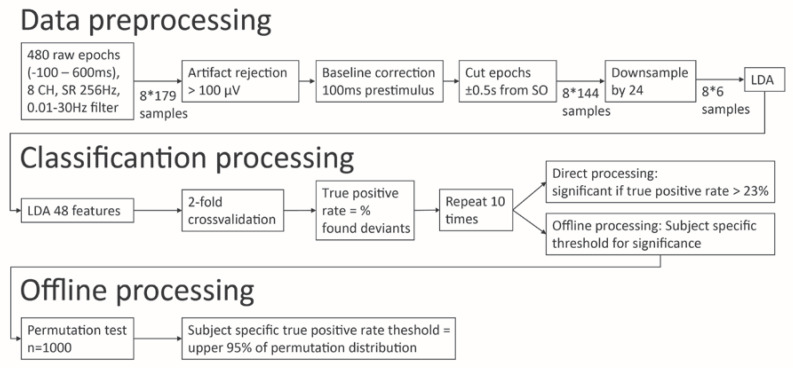
Schematic overview of data processing and classification. Data preprocessing and classification is performed right after data acquisition in order to provide feedback about the patient’s performance directly. Additional offline processing was performed to calculate a subject specific statistical threshold of the observed true positive rate.

The BCI system includes a data processing tool, consisting of automatic EEG preprocessing, artefact rejection and calculation of the true positive rate of the deviant stimulus detection (see below and Figure 1 for an overview of the data processing, or [29]).

Continuous EEG data were segmented into 700 ms-epochs, starting 100 ms before and ending 600 ms after the onset of the stimulus. Each epoch was baseline corrected using the 100 ms period preceding stimulus onset. Trials exceeding an amplitude of 100 µV were marked as artefact and excluded from the analysis. The remaining trials were averaged to compute the deviant and standard P3. The deviant and standard P3 performances were down-sampled to 6 samples by the factor of 24 with a moving average filter, resulting in a 48-features space (6 samples *8 channels). A linear discrimination analysis was used to discriminate standard and deviant trials. A two-fold cross-validation was used to evaluate the true positive rate for every subject, and was repeated ten times.

The true positive rate was calculated as the percentage of detected deviant trials over the total number of deviant trials (i.e., 60). To evaluate if the true positive rate was above chance level, which we refer here as ‘differentiated response’, two different approaches were used. In the ‘direct processing’, the significance level was set at 23% (binomial test, n = 60 trials, alpha = 0.05, for more details, see the previous studies in completely locked-in syndrome [30] and UWS [31] patients). In the ‘offline processing’, a subject-specific level of significance was computed using a permutation test (as described in [32,33], 1000 repetitions, *p* < 0.05).

### 2.3. Statistics

Statistics were performed using the statistical computing software R [34].

Chi-square statistics were used to test for group differences (Healthy participants, EMCS, MCS, UWS) in the distribution of gender and aetiology. ANOVA followed by Tukey HSD post-hoc comparisons were used to test group differences (Healthy participants, EMCS, MCS, UWS) in age and time elapsed since injury. A Welch two sample t-test was employed to test age differences between the patients and healthy participant group.

To evaluate the quality of the data processing algorithm, the differences in true positive rate between ‘direct processing’ and ‘offline processing’ were compared by means of one-sample Chi-Square test.

For further calculations, the results of the ‘offline processing’ were used. To investigate the relationship between group and ‘differentiated response’ in none, either or both paradigms, Chi square statistics of independence was calculated. For calculations that included the P3 performance in both paradigms, participants with a missing value in one of the paradigms were excluded list-wise. The Chi-square statistics were calculated for the whole sample (patients and healthy participants) and for patients only, to ensure that the results are not solely driven by the healthy participants.

In order to estimate the predictive value of diagnosis (UWS and MCS only), aetiology and time since injury on the differentiated response, a logarithmic regression model was employed for both paradigms separately. The ‘differentiated response’ (i.e., for the AEP or VTP) was used as dependent variable. Diagnosis, time since injury, aetiology, were integrated as independent variables in the logarithmic regression model.

## 3. Results

Data acquisition problems affecting either or both paradigms occurred and led to data exclusion of 18 DOC patients and 3 healthy participants. The final and complete dataset consisted of 15 UWS (51.8 ± 15.9 years, 9 males, 4 TBI), 23 MCS (42.3 ± 16.4 years, 16 males, 10 TBI) and 2 EMCS (25.0 ± 17.6 years, 2 males, 1 TBI) patients and 12 healthy participants (26.3 ± 7.2 years, 4 males).

Patients were older than healthy participants (t (34.06) = −6.21, *p* < 0.0001, 95% CI = [−27.1, −13.7]), but age did not differ amongst the different patient groups (adjusted *p*-value EMCS-MCS = 0.17; EMCS-UWS = 0.05; MCS-UWS = 0.45). There was no difference in the distribution of gender between patients and healthy participants (χ^2^ = 5.74, *p* = 0.11). More UWS than MCS patients suffered from a non-traumatic brain injury (χ^2^ = 7.73, *p* = 0.048) but no difference was found for time since injury (F (2, 36) = 1.82, *p* = 0.18).

There was evidence for a difference in the proportion of ‘differentiated responses’ in the two ways of defining the significance threshold (i.e., the ‘direct’ and ‘offline’ single subject-based threshold) for both paradigms (AEP: (χ^2^ (1, N = 52) = 11.3, *p* < 0.0001), VTP: (χ^2^ (1, N = 52) = 17.0, *p* <0.0001)).

**Figure 2:**
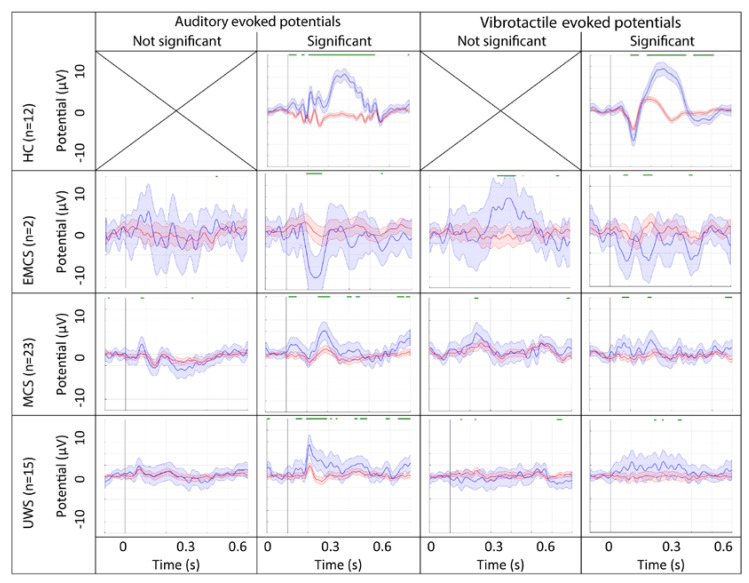
Grand average event-related potentials (ERP) at electrode Cz for the auditory and somatosensory (i.e., vibrotactile) paradigms. ERPs are grouped by diagnostic group and significance of the response. The response (and 95% confidence interval) to the standard stimulus is represented in red and the response to the deviant stimulus is represented in blue. Significant differences between the two responses, as obtained from a Mann-Whitney U test considered significant at *p* < 0.05, are presented by the green line above the ERP.

‘Direct processing’ suggested that the P3 response was significant for 28 (of 52 healthy and DOC) participants for the AEP, and for 29 (of 52 healthy and DOC) for the VTP. The ‘offline processing’ revealed a ‘differentiated response’ in the AEP paradigm for 25 (of 52 healthy and DOC) participants and in the VTP for 29 (of 52 healthy and DOC) participants (Table 1). Seven participants (3 UWS, 4 MCS) showed solely a ‘differentiated response’ during the AEP. Eleven participants (5 UWS, 6 MCS) showed solely a ‘differentiated response’ during the VTP. Eighteen participants (5 MCS, 1 EMCS, 12 healthy participants) showed a ‘differentiated response’ during both paradigms. Sixteen participants did not show a ‘differentiated response’ to either paradigm (7 UWS, 8 MCS, 1 EMCS). Grand average ERPs per diagnostic group and significance of the response are presented in Figure 2. 

**Figure 3:**
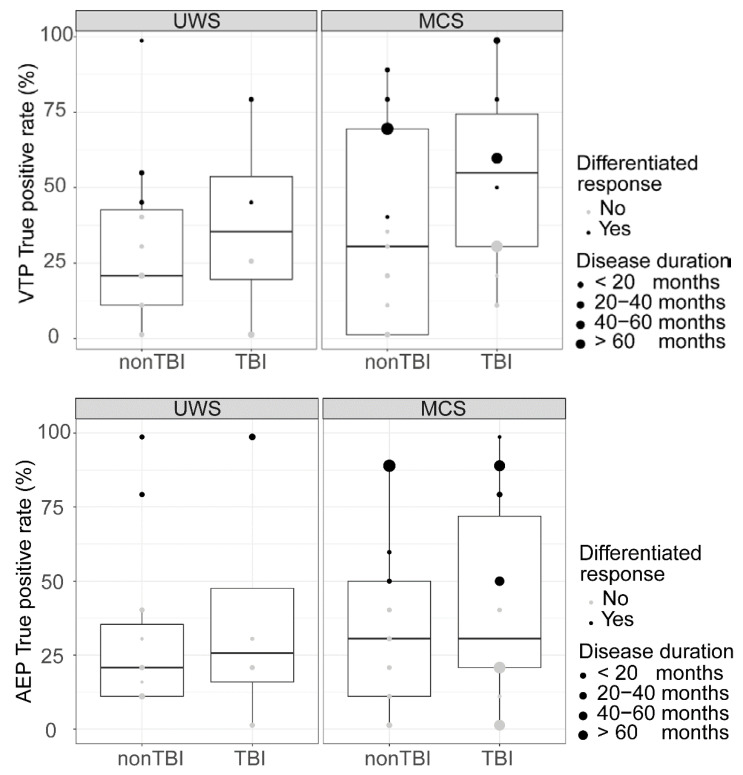
Boxplot showing the ‘differentiated response’ as obtained from offline processing and true positive rate for the auditory-evoked potentials (AEP) (**top**) and vibrotactile-evoked potentials (VTP) (**bottom**) paradigms, depending on diagnosis, aetiology and disease duration. The ‘differentiated response’ is independent from diagnosis aetiology, and disease duration.

The logarithmic regression model to predict ‘differentiated response’ in the AEP paradigm showed that neither diagnosis (OR: 0.48, 95%CI: [−2.45, 0.82]), aetiology (OR: 0.92, 95%CI: [−1.75, 1.44]) or time since injury (OR: 1.00, 95%CI: [−0.0005, 0.001]) were significant predictors (Figure 3, top). The logarithmic regression model to predict ‘differentiated response’ in the VTP paradigm did not show diagnosis (OR: 0.68, 95%CI [−1.86, 1.06]), aetiology (OR: 2.27, 95%CI [−0.61, 2.29]) or disease duration (OR: 1.00, 95%CI [−0.0009, 0.002]) to be significant predictors (Figure 3 bottom).

**Figure 4:**
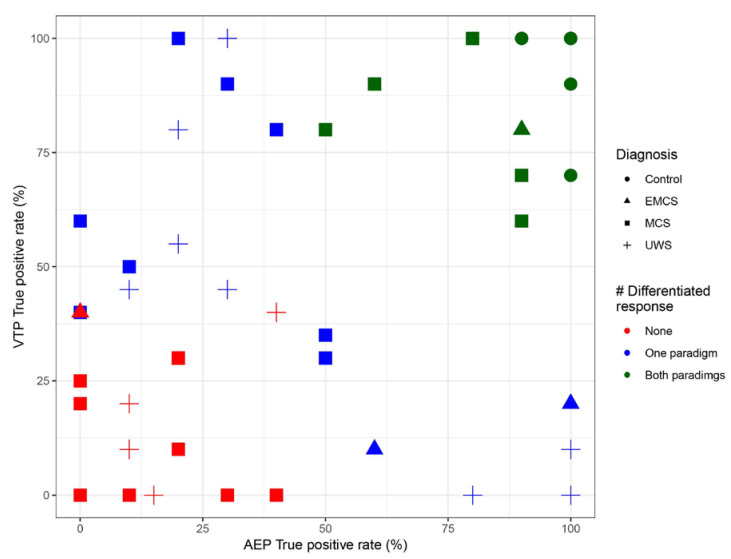
Visualisation of the true positive rate for the auditory (*x*-axis) and somatosensory (*y*-axis) paradigms. There is a correlation between the true positive rate in both paradigms, however the presence of a ‘differentiated response’ as obtained from offline processing in one paradigm is independent from the presence of a ‘differentiated response’ on the other paradigm.

The distributions of participants with a ‘differentiated response’ for the AEP and VTP were dependent on the performance of the other paradigm in healthy participants and patients together (χ^2^ (1, N = 52) = 3.95, *p* = 0.047). However, independency between the two paradigms was observed when considering patients only (χ^2^ (1, N = 40) = 0, *p* = 1, Figure 4).

## 4. Discussion

In the present study, P3-based oddball paradigms were employed to evaluate patients’ P3 performance to auditory and somatosensory stimulation as additional diagnostic measurements to complement the behavioural assessment of patients with DOC.

We first tested if the P3 performances were equally reliable during direct and offline processing, and dependent on diagnosis, aetiology and time since injury. The ‘direct’ and ‘offline’ processing yielded a different interpretation of the true positive rate (i.e., if the obtained true positive rate corresponds to a ‘differentiated response’), suggesting that caution is required when using a fixed and theoretically based threshold (in this case of 23%). The permutation-based (i.e., tailored to the patient) threshold was often higher than 23%, hence false positive results are to be expected using ‘direct’ processing. Additional ‘offline processing’ increases the validity and interpretability of the results as false positive or false negative results (both raising ethical issues [35]) are significantly reduced when a single-subject threshold is defined [33]. Even if the direct processing of ERP recordings provides a promising perspective for real-time BCI applications, its clinical application as implemented in the current system seems premature. Efforts should be intensified to improve online processing of P3 performance.

There was no evidence for a difference in the presence of ‘differentiated response’ between UWS and MCS patients, regardless of aetiology. This finding corroborates with previous research showing the presence of the P3 in different subacute and chronic states of unconsciousness [36]. Yet, a P3 performance to the own name might be observed prior to the first behavioural signs of consciousness [36] and the presence of auditory discrimination during coma is predictive for awakening [37]. However, the current study focused on patients in a sub-acute and chronic DOC in which our findings confirm previous work suggesting that the P3 performance is less informative for these patients [22,38].

Second, we tested whether the P3 performance to the auditory and tactile stimulation differed within participants to evaluate if multimodal assessment provides additional information over unimodal assessment. We evaluated whether participants showed a ‘differentiated response’ to both, one or none of the paradigms. About a quarter of the patients showed a ‘differentiated response’ to only one of the paradigms. Given that DOC patients suffer from a severe brain injury, it is possible that either stimulus is not processed efficiently. Hence, we propose that a multimodal assessment of DOC patients can account for the individual impairments in the patient group, and in this way improve the detection of potential covert abilities. Future studies should formally test this by assessing P3 performance to different modalities in patients with characterised sensory impairments (e.g., using classical auditory/somatosensory evoked potentials), and select the most promising sensory modality for every patient. Then, once above chance level true positive rate (i.e., using offline permutation-based single subject thresholds) is established, the modality with highest true positive rate could be employed in active paradigms to detect signs of covert command following, and ultimately for BCI-based communication. However, as mentioned above, for BCI applications it is crucial that the online detection of above chance-level P3 performance improves. This would ensure to establish reliable communication, avoid false positive (and false negative) results. Furthermore, supplementary neuroimaging could provide insights in the residual cognitive function and help to evaluate whether the P3 performance (and ultimately communication) fits with the patient’s residual brain function [39].

Previous EEG studies have found signs of covert command following in DOC patients. EEG-based systems for DOC patients have successfully employed motor imagery paradigms in 19% of the UWS [40] and 22% of the MCS [41] patients. A recent study reports that 75% of severely brain injured patients (including UWS, MCS and EMCS patients) were able to show command following using various active imagery paradigms [42]. Our findings built up on these results and support the need of different active paradigms to maximise the chances of establishing covert command following and potentially communication in this heterogeneous group of DOC patients.

The P3 could be used for communication purposes. However, even if not tested in this study, it is important to stress that communication should be assessed in a structured way similar to as should be done during behavioural evaluation. First, by asking the participant to reply ‘yes’ and ‘no’, then by asking simple biographical or situational oriented questions as described in the CRS-R [43] that includes a double question to which the answer is once ‘yes’ and once ‘no’. Only then it can be confirmed that communication is reliable.

A limitation of the proposed method is its requirement in recording the brain response to a sufficient number of stimuli in order to draw valid conclusions; each patient being evaluated only once with the system. However, even if the patient was positioned upright and the measurement was paused several times to avoid fatigue, fluctuations in the level of arousal could impact the assessments. Similar to behavioural assessment [5], future studies should repeat the assessment and include different ultradian and circadian time windows [44,45] to maximise the chance to detect a ‘differentiated response’ for the P3 paradigm. Another limitation of the auditory P3 paradigm is its monotonous structure. The assessment could benefit from incorporating more complex stimuli (e.g., natural voice, words, own names [46,47]), as the P3 is elicited more frequently by complex harmonic tones (e.g., musical chords, words) than by sinusoidal tones [20]. Furthermore, classical somatosensory and auditory evoked potentials could be implemented to evaluate if the top-down connections are preserved and the specific modality could be used. In order to increase the salience of the active paradigms, future studies could also use different somatosensory stimuli. A multisensory integration task could increase the patient’s involvement, and would allow studying multisensory integration. In the future, a combination of P3, motor imagery, steady state visually evoked potentials, steady state somatosensory evoked potentials paradigms and other BCIs should be applied (with stimulation parameters adjustable by the user) and compared within participants to evaluate the effectiveness of every paradigm for the assessment of command following and (ultimately) communication in DOC patients. Indeed, monitoring multiple sensory channels recruits a more extended brain network than either modality-specific network [48], and the information transfer rate during an auditory-tactile P3 session is about 50% higher than during either unimodal session [49]. Thus, the cortical response seems to be spatially extended and of higher amplitude, which should facilitate the quantification of the P3 performance in general and in DOC patients in specific.

## 5. Conclusions

In conclusion, we here show that even if the P3 performance using AEP and VTP does not depend on diagnosis or aetiology, some patients showed a ‘differentiated response’ to only one of the paradigms. This has important implications for future BCI technologies as it supports the need for development of tools involving multiple, and not only auditory, modalities.

## Figures and Tables

**Table 1:** Clinical data (gender, age in years, time post injury in months, aetiology, CRS-R subscores) and P3 true positive rate for all participants (HC, EMCS, MCS, UWS) enrolled in the study after direct processing and after offline processing of the two paradigms (AEP, VTP). True positive rate in bold represents that is is above chance level (i.e. ‘differentiated responses’).

						Direct		Offline	
Group	Gender	Age	Time Since Injury	Aetiology	CRS-R Subscores	AEP	VTP	AEP	VTP
HC	F	28		nonTBI		**100**	**100**	**100**	**100**
HC	F	23		nonTBI		**100**	**100**	**100**	**100**
HC	M	23		nonTBI		**90**	**100**	**100**	**100**
HC	F	21		nonTBI		**100**	**100**	**100**	**100**
HC	F	24		nonTBI		**100**	**80**	**100**	**90**
HC	F	22		nonTBI		**100**	**100**	**90**	**100**
HC	F	27		nonTBI		**100**	**100**	**100**	**100**
HC	M	24		nonTBI		**100**	**100**	**80**	**100**
HC	F	25		nonTBI		**100**	**100**	**100**	**70**
HC	F	24		nonTBI		**100**	20	**90**	**100**
HC	M			nonTBI		**100**	**100**	**90**	**100**
HC	M	48		nonTBI		**100**	**100**	**100**	**100**
EMCS	M	24		nonTBI	1,3,2,1,0,2	20	**60**	0	40
EMCS	M	26	3.9	TBI	3,5,4,1,0,2	**70**	**90**	**90**	**80**
MCS	M	66	4.5	TBI	2,3,5,3,1,2	10	**70**	20	10
MCS	F	43	2.6	nonTBI	2,3,6,2,1,2	**30**	20	**60**	10
MCS	F	19	4.2	nonTBI	1,0,5,1,0,1	20	20	40	0
MCS	M	25	4.8	nonTBI	1,0,5,1,0,2	20	**70**	**50**	**80**
MCS	F	51	5.1	nonTBI	2,3,2,2,1,2	**60**	**55**	30	**90**
MCS	M	63	2.1	nonTBI	2,2,0,1,0,1	10	10	0	**40**
MCS	M	47	3.5	TBI	2,3,2,1,0,2	**60**	**100**	40	**80**
MCS	M	58	2.4	nonTBI	3,1,5,2,1,2	0	**30**	**50**	35
MCS	M	53	3.0	nonTBI	2,3,2,2,0,2	0	**40**	**60**	**90**
MCS	M	61	4.3	nonTBI	2,3,2,1,0,1	**100**	0	10	0
MCS	M	56	5.0	nonTBI	2,1,5,2,1,1	20	20	0	20
MCS	M	34	1.7	TBI	4,5,6,1,1,2	20	10	**100**	20
MCS	M	52	1.9	TBI	3,3,5,1,1,1	0	20	10	**50**
MCS	M	64	55.9	TBI	3,5,6,2,1,2	0	**90**	0	**60**
MCS	M	18	7.8	TBI	3,3,5,2,0,1	20	**100**	**80**	**100**
MCS	M	55	68.7	TBI	2,0,5,1,0,1	**30**	10	20	30
MCS	M	22	39.2	TBI	1,3,1,1,0,1	**50**	20	**50**	30
MCS	F	41	79.0	nonTBI	3,0,1,1,0,1	**40**	**60**	**90**	**70**
MCS	M	20	13.7	TBI	3,3,5,2,0,2	10	**100**	20	**100**
MCS	M	57	7.8	nonTBI	1,1,1,1,0,1	20	**25**	0	0
MCS	F	49	3.9	nonTBI	2,1,1,2,0,2	**30**	0	30	0
MCS	F	47	3.9	nonTBI	1,3,5,1,0,1	20	10	20	30
MCS	F	40	58.9	TBI	0,3,2,1,0,2	**60**	**100**	**90**	**60**
UWS	F	33	10.5	nonTBI	2,1,1,1,0,2	**30**	**40**	10	20
UWS	M	46	4.9	TBI	1,0,1,1,0,2	0	10	20	**80**
UWS	F	27	2.2	TBI	2,1,1,1,0,2	10	**40**	30	**45**
UWS	M	59	2.0	nonTBI	2,0,2,1,0,2	10	0	10	10
UWS	M	65	5.0	nonTBI	1,0,2,1,0,2	10	0	40	40
UWS	F	54	4.3	nonTBI	1,0,1,1,0,2	0	**50**	10	**45**
UWS	M	50	5.6	TBI	2,2,2,1,0,2	10	20	0	25
UWS	M	57	3.7	nonTBI	0,0,5,1,0,0	**30**	10	20	30
UWS	M	57	1.5	nonTBI	2,1,2,1,0,1	**80**	0	30	**100**
UWS	M	65	4.9	nonTBI	1,1,1,1,0,1	**100**	0	**100**	10
UWS	M	56	5.9	nonTBI	1,1,1,1,0,1	10	**75**	20	**55**
UWS	F	46	5.9	nonTBI	0,0,1,1,0,2	**70**	0	**80**	0
UWS	F	71	1.3	nonTBI	0,0,1,1,0,1	10	10	15	0
UWS	F	32	12.7	TBI	1,0,1,1,0,2	**60**	10	**100**	0
UWS	M	59	2.0	nonTBI	2,1,1,1,0,2	20	0	10	10

HC = healthy control, EMCS = emerged from minimally conscious state, MCS = minimally conscious state, UWS = unresponsive wakefulness syndrome, M = male, F = female, TBI = traumatic brain injury; nonTBI = non-traumatic brain injury, CRS-R = coma recovery scale-revised presenting auditory, visual, motor, verbal, communication, arousal subscores, Direct = direct processing provided by a theoretical threshold of 23%, Offline = further offline processing taking the individual threshold based on permutation tests, AEP = auditory evoked potential, VTP = vibrotactile potentials. Bold numberdenotes significant true positive rate.
